# MIRU-VNTR Typing of Atypical Mycobacteria Isolated from the Lymph Nodes of Slaughtered Pigs from Poland

**DOI:** 10.3390/pathogens11050495

**Published:** 2022-04-21

**Authors:** Marta Majchrzak, Aleksandra Kaczmarkowska, Anna Didkowska, Sylwia Brzezińska, Blanka Orłowska, Daniel Klich, Ewa Augustynowicz-Kopeć, Krzysztof Anusz, Paweł Parniewski

**Affiliations:** 1 Institute of Medical Biology, Polish Academy of Sciences, Lodowa 106, 93-232 Lodz, Poland; mmajchrzak@cbm.pan.pl; 2 Department of Food Hygiene and Public Health Protection, Institute of Veterinary Medicine, Warsaw University of Life Sciences (SGGW), Nowoursynowska 159 c, 02-787 Warsaw, Poland; anna_didkowska@sggw.edu.pl (A.D.); blanka_orlowska@sggw.edu.pl (B.O.); krzysztof_anusz@sggw.edu.pl (K.A.); 3 Department of Microbiology, National Tuberculosis Reference Laboratory, National Tuberculosis and Lung Diseases Research Institute, Płocka 26, 01-138 Warsaw, Poland; s-brzezinska@wp.pl (S.B.); e.kopec@igichp.edu.pl (E.A.-K.); 4 Department of Animal Genetics and Conservation, Institute of Animal Sciences, University of Life Sciences (SGGW), Ciszewskiego 8, 02-786 Warsaw, Poland; daniel_klich@sggw.edu.pl

**Keywords:** food safety, lymph nodes, MIRU-VNTR, *Mycobacterium avium*, pig slaughter, pork

## Abstract

No regulations currently require the excision of lymph nodes from pig carcasses or the thermal processing of pork before consumption. Therefore, the presence of anatomopathological lesions with signs of coagulation necrosis in lymph nodes from pigs during post-mortem inspection is concerning, as is the increasing incidence of mycobacteriosis in humans. Therefore, the aim of the present study is to verify whether mycobacteria can be isolated from tuberculous-like lesions in mandibular lymph nodes in slaughtered pigs, and whether further molecular analysis based on MIRU-VNRT, used to identify mycobacteria from the *Mycobacterium avium* complex, can indicate zoonotic potential. Forty of the fifty isolates from the lymph nodes with signs of coagulation necrosis were classified as *Mycobacterium avium* complex. MIRU-VNTR analysis allowed for the isolation of six strains, one of which was classified as *M. avium* subsp. *paratuberculosis* (MAP). Our findings confirm the presence of atypical mycobacteria in the lymph nodes of slaughtered pigs. While the isolated strains (other than MAP) do not pose a significant or direct health risk to consumers, further research and monitoring are necessary. Atypical mycobacteria can cause a wide range of diseases in children and compromised adults, and often show resistance to many classes of antibiotics, including those used to treat tuberculosis.

## 1. Introduction

The non-tuberculous mycobacteria (NTM) vary considerably. However, while most are environmental microorganisms, some of them, especially those of the *Mycobacterium avium* complex (MAC), can act as opportunistic pathogens [1], being responsible for a range of infections in both animals and humans. In pigs, the primary etiological agents of mycobacterial infection are four subspecies of *M. avium*: *M. avium* subsp. *avium* (MAA), *M. avium* subsp. *hominissuis* (MAH), *M. avium* subsp. *silvaticum* (MAS), and *M. avium* subsp. *paratuberculosis* (MAP) [2]. Of these, MAA and MAH have the highest epidemiological importance in pigs. MAA is an etiologic agent of avian tuberculosis, with the feces of infected birds being the primary source of infection [3,4,5]. 

While most publications on mycobacterial infection in pigs have generally focused on MAH, some isolates from granulomatous lesions in pigs are caused by MAA [6]. The genetic differences between MAH strains isolated from humans and pigs are unclear [7]. 

MAC bacteria are typically identified or genotyped using various molecular techniques, such as restriction fragment length polymorphism (RFLP), pulsed-field gel electrophoresis (PFGE), mycobacterial interspersed repetitive units–variable number of tandem repeats (MIRU-VNRT), repetitive element sequence-based PCR (rep-PCR), or trinucleotide repeat sequence-based PCR (TRS-PCR) [4,8,9,10,11,12]. Not all methods allow differentiation between strains belonging to the same subspecies. For example, RFLP is better suited to differentiating between subtypes of mycobacteria as it is based on polymorphisms present in fragments created by restriction enzymes for insertion sequences (IS) IS 1311, IS 1245, and IS 900 [13,14,15].

Genotyping is a valuable tool in epidemiological research and can play an important role in disease prevention by revealing the source of infection and the route of transmission. The most widely used typing method for *M. avium* is MIRU-VNTR [16]. For genotyping *M. avium* isolates, MIRU-VNTR assays are based on eight loci with high discriminant indices: MIRU 292, MIRU X3, VNTR 25, VNTR 47, VNTR 3, VNTR 7, VNTR 10, and VNTR 32 [17]. The aim of the present study was to determine the value of MIRU-VNTR analysis in evaluating the genetic diversity of *M. avium* strains isolated from slaughtered pigs in Poland. 

## 2. Results

### 2.1. Mycobacterial Analysis and Species Designation

The isolated strains were classified as *M. avium* based on the appearance of single colonies on differentiating media. To confirm the presence of MAC strains, IS analysis was performed. Out of 50 strains, 39 showed the presence of IS 901 and IS 1245, none of them showed the presence of IS 900.

### 2.2. MIRU-VNTR Analysis

Six MIRU-VNTR patterns were identified in 40 of the tested isolates (Table 1). One case, with the numerical code 22331218, corresponded to a record present in the MAC-INMV-SSR database (sample number 6): *M. avium* subsp. *paratuberculosis*. As the other five numerical codes (22131227, 23131227, 24131227, 25131227, 26131227) were not identified, they were added to the database. No MLVA patterns were identified for the remaining cases.

## 3. Discussion

**Figure 1:**
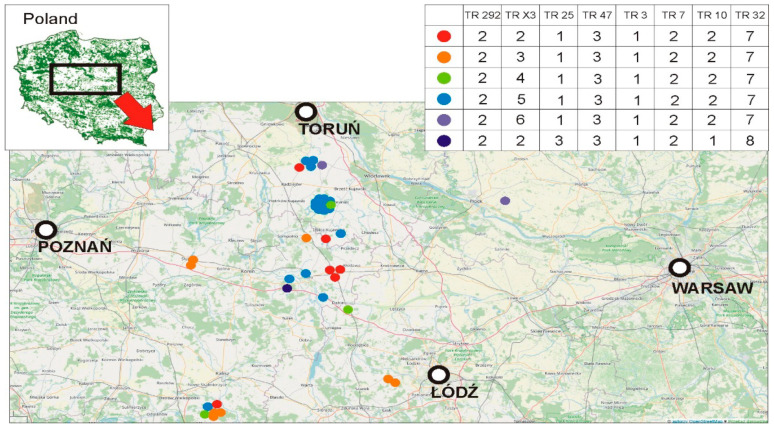
The geographical origin of the 40 MIRU-VNTR *Mycobacterium avium* profiles included in the study.

Our paper presents new data regarding the molecular characteristics of MAC present in the region. These findings provide an overview of the genetic diversity of these MAC strains and their geographical distribution (Figure 1).

The results showed that four of the eight studied loci (TR X3, TR 25, TR 10, and TR 32) were polymorphic, while the others (TR 292, TR 47, TR 3, and TR 7) had the same repeat count in all tested strains. This suggests that the four polymorphic MIRU-VNTR loci (TR x3, TR 25, TR 10, and TR 32) may have the same discriminatory power as all eight studied loci together. Additionally, only one locus differed between isolates outside of *M. avium* subsp. *paratubeculosis*.

The TR X3 locus demonstrated the most significant allelic variability, as indicated by an h-value of 0.69, calculated using the Nei index. The discriminant power of the method (D) was found to be 0.7058, which is consistent with other publications [18,19]. Among all the obtained MIRU-VNRT profiles, apart from IMNV 78 (MAP), the number of tandem repeats differed only within the TR X3 locus.

None of the tested strains demonstrated the IS 900 sequence; however, all strains, with the exception of *M. avium* subsp. *paratuberculosis*, contained the IS 901 sequence. Although it is surprising that the IS 900 sequence was absent from the INMV 78 profile, previous research suggests the existence of a small group of *M. avium* subsp. *paratuberculosis* lacking the IS 900 element or possibly possessing a different insertion sequence [20]. Moreover, it cannot be ruled out that minor genetic changes, such as SNPs or minor deletions, made it impossible to produce a PCR product.

The IS 1245 sequence was present in all of the 39 *M. avium* spp. strains, but it was not found in MAP—which is consistent with the obvious [21]. 

Despite the assignment of strain number 6 by the database: http://mac-inmv.tours.inra.fr/index.php?p=fa_ident (accessed on 2 February 2022) to the MAP subspecies, additional analyses of F57 or IS 1311 sequence testing would be required to confirm that the strain actually belongs to this subspecies.

In the animals infected with *M. avium*, the most common location of macroscopic lesions was in the lymph nodes; however, other lesions were found in the liver and lungs. Our findings indicate that *M. avium* may be present in tissues that do not display any visible changes [6], and therefore, that such meat may be included in the human food chain. In this context, minced meat seems to be particularly dangerous, as it may contain lymph nodes [22]. As mycobacteria can also survive heat treatment and some disinfection treatments (glutaraldehyde [23], chlorhexidine [24]), the presence of mycobacteria in food is a potential biological hazard that should be monitored and prevented. These findings have been confirmed in a number of previous studies which have also described the detection of mycobacteria in animal tissues, confirming that food of animal origin can act as a vehicle for transmitting mycobacteria to humans [25].

The incidence of NTM disease in humans has increased dramatically over the past thirty years worldwide [26], and the majority of human NTM infections have been attributed to the *M. avium* complex [26]. Many reports also suggest that MAP may be an etiological factor in Crohn’s disease in humans [27]; however, it should be noted that these patients often fail to show improvement after anti-tuberculosis therapy [28].

The MIRU-VNTR offers the advantages of high reproducibility and the need for only a small amount of DNA as a sample [17,29]. However, previous studies have shown that VNTR typing can misjudge strain diversity and origin. In addition, it can also overestimate or underestimate the relationship between strains due to the instability of some repeating elements in the genome and the presence of homoplasy [17]. Homoplasy is the occurrence of genotypes that are identical by state but not by descent; these can arise by various means, including convergent and reverse evolution and horizontal gene transfer [30,31]. Significantly greater discriminatory power can be achieved by using a combination of different typing techniques, such as IS 900 RFLP with MIRU-VNTR analysis, which gives very good results [18,29].

Our study used eight MIRU-VNTR loci described by Thibault et al.: TR292 and TRX3 and TR 25, 47, 3, 7, 10, and 32 [18]. From the 50 tested samples, six MIRU-VNTR profiles were obtained, and these were compared with the profiles collected in the MAC-INMV database (http://mac-inmv.tours.inra.fr/) (accessed on 2 February 2022). One of the profiles corresponded to *M. avium paratuberculosis* (IMNV 78), which had been previously isolated and described in patients with suspected tuberculosis in Brazil [32]. However, the five remaining profiles were not in the database. The strains belonging to the IMNV 245 and INMV 244 profiles had not previously been added to the database; however, they had previously been isolated from pig lymph nodes in Argentina and identified as *Mycobacterium avium* subsp. *avium* [4].

In the present study, pigs from the same farm always demonstrated the same MIRU-VNTR profile. In addition, identical MIRU-VNTR profiles were also obtained from pigs from different farms (Figure 1).

In the longer term, it would be valuable to identify any differences between the isolated granulomatous lesions of these five strains, as well as the size and characteristics of the central necrosis, and the extent of the lesion distribution.

## 4. Materials and Methods

### 4.1. The Origin of Material and Culture 

The material consisted of submandibular lymph nodes of pigs in Poland, which had previously been archived in the laboratory. The locations of the farms from which the nodes were collected are shown in Figure 1. Pigs were not moved between farms. 

The acid-fast mycobacteria were isolated in accordance with the recommendations of the Reference Microbiological Laboratory of the National Research Institute—National Veterinary Institute in Puławy. The material was shredded with sterile scissors and placed in bags with a filtering membrane (BagPage^®^ 100). In the next step, the material was immersed in a 5% oxalic acid solution (POCH, Zabrze, Poland), then homogenized in a stomacher for three minutes at a rate of 12 strokes/second. The resulting solution was poured into tubes, which were incubated for 10–15 min at 37 °C, and then centrifuged for 10 min at 1500× *g*. The supernatant was then removed, and sterile 0.9% NaCl was added up to the maximum volume of the tube. The tubes were shaken by hand and centrifuged for 10 min at 1500× *g*, this operation was repeated twice. The sediment was inoculated onto solid Löwenstein–Jensen (MERCK, Darmstadt, Germany) and Stonebrink (MERCK, Germany) media for the cultivation of mycobacteria. The cultures were incubated at 37 °C for 12 weeks. The media was checked for mycobacterial colony growth once a week. No growth after 12 weeks was considered a negative result.

### 4.2. DNA Isolation

DNA was extracted from Löwenstein–Jensen and Stonebrink media isolates using the thermal method (95 °C, 30 min). Briefly, an inoculum loop filled with mycobacterial colonies isolated on Löwenstein–Jensen or Stonebrink media was suspended in 150 μL of water, and then incubated for 30 min in a thermoblock at 95 °C. After incubation, the tubes were centrifuged for five minutes at 15,000× *g*. The supernatant was used for testing.

### 4.3. Strain Identification 

The isolated strains were identified to species level using GenoType^®^Mycobacterium CM (Hain Lifescience, Nahren, Germany). The test was performed according to the manufacturer’s instructions.

### 4.4. IS901, IS900, and IS1245 Identification

Fifty isolates were subjected to IS 901, IS 900, and IS 1245 analysis according to the previously described method with modifications [33,34,35,36,37]. This method is used for the rapid identification of MAC species. For IS 900 and IS 901, the PCR protocol was optimized, and the reaction was performed in a total volume of 50 µL containing the following mixture: 20 ng of DNA, 5× Taq polymerase reaction buffer (Invitrogen by Life Technologies, Carlsbad, CA, USA), 1 U Taq polymerase (Invitrogen by Life Technologies, Waltham, MA, USA), 1.5 mM of MgCl2, 200 µM of each deoxynucleotide, 2 µL dimethyl sulfoxide (DMSO), and 2 mM of each primer. The following conditions were used: an initial denaturation step at 94 °C for 5 min, followed by 30 cycles of denaturation at 94 °C for 30 s, annealing at 58 °C for 30 s, and extension at 72 °C for 30 s; and this was followed by a final extension step at 72 °C for 7 min. For IS1245, the PCR reaction was performed in a total volume of 50 µL containing the following mixture: 20 ng of DNA, 25 µL Platinum Multiplex PCR Master Mix 2x (Applied Biosystems, Carlsbad, CA, USA) and 50 nM each primer. The following conditions were used: an initial denaturation step at 95 °C for 15 min, followed by 28 cycles of denaturation at 95 °C for 30 s, annealing at 60 °C for 90 s and extension at 72 °C for 1 min.; and this was followed by a final extension step at 72 °C for 2 min. All reactions were performed using a T3000 thermal cycler (Analytik Jena, Jena, Germany).

Electrophoresis was performed at 70 V (2.4 V/cm) until the dye (bromophenol blue) reached 6 cm from the wells. The gels were then stained in an ethidium bromide (EtBr) solution (0.5 µg/mL) for 10 min and destained in water for another 10 min. The gels were visualized under UV light using a FluorChem 8800 system with Alpha EaseFC v. 3.1.2 software (AlphaInnotech, San Leandro, CA, USA). For all analyzed IS types, a 100 bp Plus ladder size marker from MBI Fermentas was used. The predicted sizes of PCR fragments for IS900 and IS901 are 389 bp and 262 bp, respectively.

### 4.5. MIRU-VNTR Identification

The MIRU-VNTR method used in the present study is a simple tool for genotyping mycobacteria and detecting possible phylogenetic relationships between strains. The isolates were subjected to multi-locus variable-number tandem repeat analysis (MLVA), according to the eight variable-number tandem-repeat (VNTR) locus scheme proposed by Thibault et al. [18]. Primer sequences for the amplification of the TR292, TRX3, TR25, TR47, TR3, TR7, TR10, and TR32 loci were selected from Cochard et al. [38] (Table 1). The PCR was optimized and performed in a total volume of 25 µL consisting of the following mixture: 20 ng of DNA, 5× Taq polymerase reaction buffer (Invitrogen by Life Technologies, CA, USA), 1 U Taq polymerase (Invitrogen by Life Technologies, Waltham, MA, USA), 1.5 mM of MgCl2, 200 µM of each deoxynucleotide, 6% dimethyl sulfoxide (DMSO), 1 M betaine solution and 0.2 µM of each primer. The reactions were performed using a T3000 thermal cycler (Analytik Jena, Jena, Germany) under the following conditions: initial denaturation at 95 °C for 3 min, followed by 30 cycles of denaturation at 95 °C for 30 s, annealing (at 55 °C for TR32; 58 °C for TR292, TRX3, TR25; 60 °C for TR3, TR7, TR10; 64 °C for TR47) for 30 s and extension at 72 °C for 1 min, and then final extension at 72 °C for 5 min.

The products were subjected to electrophoresis at 70 V (2.4 V/cm) until the dye (bromophenol blue) reached 6 cm from the wells. The gels were then stained in an ethidium bromide (EtBr) solution (0.5 µg/mL) for 10 min and destained in water for another 10 min. They were then visualized under UV light using a FluorChem 8800 system with Alpha EaseFC v. 3.1.2 software (AlphaInnotech, San Leandro, CA, USA). 

For all analyzed VNTRs, a 100 bp Plus ladder size marker from MBI Fermentas was used. The size of each amplicon was measured with BioNumerics (version 4.6) software (Applied-Maths, Saint-Martens-Latem, Belgium). The sizes of the PCR products were used to assess the number of motif repeats (Table 2). The numerical codes were compared with those registered in the MAC-INMV-SSR database (http://mac-inmv.tours.inra.fr/) (accessed on 2 February 2022). 

## 5. Conclusions

MAC bacilli are present in altered lymph nodes in slaughtered pigs. However, the isolated mycobacteria do not appear to pose a risk to pork consumers as the tested strains have not been previously isolated from humans, but they may just not be known to compromised hosts. However, their zoonotic potential cannot be ruled out, and further research should be continued on a larger scale.

## Figures and Tables

**Table 1:** MIRU-VNTR and IS profiles of isolated strains.

Isolate	Number of Copies MIRU-VNTR Region								Sub-Species Assignment	IS901	IS900	IS1245
	TR 292	TR x3	TR 25	TR 47	TR 3	TR 7	TR 10	TR 32				
**5**	**2**	**2**	**1**	**3**	**1**	**2**	**2**	**7**	***M. avium* spp.**	**+**	**−**	**+**
**7**	**2**	**2**	**1**	**3**	**1**	**2**	**2**	**7**	***M. avium* spp.**	**+**	**−**	**+**
**9**	**2**	**2**	**1**	**3**	**1**	**2**	**2**	**7**	***M. avium* spp.**	**+**	**−**	**+**
**16**	**2**	**2**	**1**	**3**	**1**	**2**	**2**	**7**	***M. avium* spp.**	**+**	**−**	**+**
**17**	**2**	**2**	**1**	**3**	**1**	**2**	**2**	**7**	***M. avium* spp.**	**+**	**−**	**+**
**23**	**2**	**2**	**1**	**3**	**1**	**2**	**2**	**7**	***M. avium* spp.**	**+**	**−**	**+**
**41**	**2**	**3**	**1**	**3**	**1**	**2**	**2**	**7**	***M. avium* spp.**	**+**	**−**	**+**
**42**	**2**	**3**	**1**	**3**	**1**	**2**	**2**	**7**	***M. avium* spp.**	**+**	**−**	**+**
**43**	**2**	**3**	**1**	**3**	**1**	**2**	**2**	**7**	***M. avium* spp.**	**+**	**−**	**+**
**75**	**2**	**3**	**1**	**3**	**1**	**2**	**2**	**7**	***M. avium* spp.**	**+**	**−**	**+**
**89**	**2**	**3**	**1**	**3**	**1**	**2**	**2**	**7**	***M. avium* spp.**	**+**	**−**	**+**
**109**	**2**	**3**	**1**	**3**	**1**	**2**	**2**	**7**	***M. avium* spp.**	**+**	**−**	**+**
**111**	**2**	**3**	**1**	**3**	**1**	**2**	**2**	**7**	***M. avium* spp.**	**+**	**−**	**+**
**112**	**2**	**3**	**1**	**3**	**1**	**2**	**2**	**7**	***M. avium* spp.**	**+**	**−**	**+**
**79**	**2**	**4**	**1**	**3**	**1**	**2**	**2**	**7**	***M. avium* spp.**	**+**	**−**	**+**
**80**	**2**	**4**	**1**	**3**	**1**	**2**	**2**	**7**	***M. avium* spp.**	**+**	**−**	**+**
**20ab**	**2**	**4**	**1**	**3**	**1**	**2**	**2**	**7**	***M. avium* spp.**	**+**	**−**	**+**
**2**	**2**	**5**	**1**	**3**	**1**	**2**	**2**	**7**	***M. avium* spp.**	**+**	**−**	**+**
**13ab**	**2**	**5**	**1**	**3**	**1**	**2**	**2**	**7**	***M. avium* spp.**	**+**	**−**	**+**
**14**	**2**	**5**	**1**	**3**	**1**	**2**	**2**	**7**	***M. avium* spp.**	**+**	**−**	**+**
27 *	**2**	**5**	**1**	**3**	**1**	**2**	**2**	**7**	***M. avium* spp.**	**+**	**−**	**+**
30 **	**2**	**5**	**1**	**3**	**1**	**2**	**2**	**7**	***M. avium* spp.**	**+**	**−**	**+**
**35**	**2**	**5**	**1**	**3**	**1**	**2**	**2**	**7**	***M. avium* spp.**	**+**	**−**	**+**
**44**	**2**	**5**	**1**	**3**	**1**	**2**	**2**	**7**	***M. avium* spp.**	**+**	**−**	**+**
**47**	**2**	**5**	**1**	**3**	**1**	**2**	**2**	**7**	***M. avium* spp.**	**+**	**−**	**+**
**48**	**2**	**5**	**1**	**3**	**1**	**2**	**2**	**7**	***M. avium* spp.**	**+**	**−**	**+**
**66**	**2**	**5**	**1**	**3**	**1**	**2**	**2**	**7**	***M. avium* spp.**	**+**	**−**	**+**
**82**	**2**	**5**	**1**	**3**	**1**	**2**	**2**	**7**	***M. avium* spp.**	**+**	**−**	**+**
**86**	**2**	**5**	**1**	**3**	**1**	**2**	**2**	**7**	***M. avium* spp.**	**+**	**−**	**+**
**87**	**2**	**5**	**1**	**3**	**1**	**2**	**2**	**7**	***M. avium* spp.**	**+**	**−**	**+**
**88**	**2**	**5**	**1**	**3**	**1**	**2**	**2**	**7**	***M. avium* spp.**	**+**	**−**	**+**
**91**	**2**	**5**	**1**	**3**	**1**	**2**	**2**	**7**	***M. avium* spp.**	**+**	**−**	**+**
**96**	**2**	**5**	**1**	**3**	**1**	**2**	**2**	**7**	***M. avium* spp.**	**+**	**−**	**+**
**99**	**2**	**5**	**1**	**3**	**1**	**2**	**2**	**7**	***M. avium* spp.**	**+**	**−**	**+**
**102**	**2**	**5**	**1**	**3**	**1**	**2**	**2**	**7**	***M. avium* spp.**	**+**	**−**	**+**
**104**	**2**	**5**	**1**	**3**	**1**	**2**	**2**	**7**	***M. avium* spp.**	**+**	**−**	**+**
**108**	**2**	**5**	**1**	**3**	**1**	**2**	**2**	**7**	***M. avium* spp.**	**+**	**−**	**+**
**90**	**2**	**6**	**1**	**3**	**1**	**2**	**2**	**7**	***M. avium* spp.**	**+**	**−**	**+**
**100**	**2**	**6**	**1**	**3**	**1**	**2**	**2**	**7**	***M. avium* spp.**	**+**	**−**	**+**
**6**	**2**	**2**	**3**	**3**	**1**	**2**	**1**	**8**	***M.a. paratuberculosis***	**−**	**−**	**−**
**3**	**not *M. avium* spp.**									**−**	**−**	**−**
**73**	**not *M. avium* spp.**									**−**	**−**	**−**
**78**	**not *M. avium* spp.**									**−**	**−**	**−**
**85**	**not *M. avium* spp.**									**−**	**−**	**−**
**92**	**not *M. avium* spp.**									**−**	**−**	**−**
**95**	**not *M. avium* spp.**									**−**	**−**	**−**
**97**	**not *M. avium* spp.**									**−**	**−**	**−**
**107**	**not *M. avium* spp.**									**−**	**−**	**−**
**113**	**not *M. avium* spp.**									**−**	**−**	**−**
124 ***	**not *M. avium* spp.**									**−**	**−**	**−**

All strains were isolated from pigs from submandibular lymph node with lesions, except strains: 27 *—mediastinal lymph node, with lesions. 30 **—lymph node of the hilum of the liver, with lesions. 124 ***—sub-mandibular lymph node, no lesions. Sub-species were identified using the database: http://mac-inmv.tours.inra.fr/index.php?p=fa_ident (accessed on 2 February 2022).

**Table 2:** The sizes of motif core and both the 5′- and 3′-flanking sequences.

MIRU-VNRT	Repeated Motif (bp)	Flanking Sequences (bp)
TR292	53	141
TRX3	53	90
TR25	58	193
TR47	35	112
TR3	27	146
TR7	22	159
TR10	55	198
TR32	18	143

## Data Availability

Not applicable.
